# Oxytocin inhibits lipopolysaccharide-induced inflammation in microglial cells and attenuates microglial activation in lipopolysaccharide-treated mice

**DOI:** 10.1186/s12974-016-0541-7

**Published:** 2016-04-13

**Authors:** Lin Yuan, Song Liu, Xuemei Bai, Yan Gao, Guangheng Liu, Xueer Wang, Dexiang Liu, Tong Li, Aijun Hao, Zhen Wang

**Affiliations:** Department of Physiology, Shandong University School of Medicine, 44#, Wenhua Xi Road, Jinan, Shandong 250012 People’s Republic of China; Department of Medical Psychology, Shandong University School of Medicine, 44#, Wenhua Xi Road, Jinan, Shandong 250012 People’s Republic of China; Key Laboratory of the Ministry of Education for Experimental Teratology, Shandong Provincial Key Laboratory of Mental Disorders, Department of Histology and Embryology, Shandong University School of Medicine, 44#, Wenhua Xi Road, Jinan, Shandong 250012 People’s Republic of China

**Keywords:** Oxytocin, Lipopolysaccharide, Neuroinflammation, Microglial activation

## Abstract

**Background:**

Overactivated microglia is involved in various kinds of neurodegenerative diseases. Suppression of microglial overactivation has emerged as a novel strategy for treatment of neuroinflammation-based neurodegeneration. In the current study, anti-inflammatory effects of oxytocin (OT), which is a highly conserved nonapeptide with hormone and neurotransmitter properties, were investigated in vitro and in vivo.

**Methods:**

BV-2 cells and primary microglia were pre-treated with OT (0.1, 1, and 10 μM) for 2 h followed by LPS treatment (500 ng/ml); microglial activation and pro-inflammatory mediators were measured by Western blot, RT-PCR, and immunofluorescence. The MAPK and NF-*κ*B pathway proteins were assessed by Western blot. The intracellular calcium concentration ([Ca^2+^]i) was determined using Fluo2-/AM assay. Intranasal application of OT was pre-treated in BALB/C mice (adult male) followed by injected intraperitoneally with LPS (5 mg/kg). The effect of OT on LPS-induced microglial activation and pro-inflammatory mediators was measured by Western blot, RT-PCR, and immunofluorescence in vivo.

**Results:**

Using the BV-2 microglial cell line and primary microglia, we found that OT pre-treatment significantly inhibited LPS-induced microglial activation and reduced subsequent release of pro-inflammatory factors. In addition, OT inhibited phosphorylation of ERK and p38 but not JNK MAPK in LPS-induced microglia. OT remarkably reduced the elevation of [Ca^2+^]_i_ in LPS-stimulated BV-2 cells. Furthermore, a systemic LPS-treated acute inflammation murine brain model was used to study the suppressive effects of OT against neuroinflammation in vivo. We found that pre-treatment with OT showed marked attenuation of microglial activation and pro-inflammatory factor levels.

**Conclusions:**

Taken together, the present study demonstrated that OT possesses anti-neuroinflammatory activity and might serve as a potential therapeutic agent for treating neuroinflammatory diseases.

## Background

Neuroinflammation mediated by glial activation is identified as a common character during progression of many neurodegenerative diseases, including Alzheimer’s disease (AD) and Parkinson’s disease (PD) [1, 2]. Microglia, the resident immune cells of the central nervous system (CNS), is considered to play a key role in regulating neurotoxicity mediated by inflammatory response. Microglial activation can be induced by lipopolysaccharide (LPS), interferon (IFN)-γ, or β-amyloid and results in overproduction of inflammatory cytokines. These inflammatory mediators, such as tumor necrosis factor-α (TNF-α), interleukin (IL) -6, IL-1β, glutamate, nitric oxide, and reactive oxygen species, can collectively lead to neuronal damage, resulting in the progress of neurodegenerative diseases [3]. During this process, ramified resting microglia undergo morphological transformations including deramification, process shortening and thickening, and finally development into its activated amoeboid form [4]. Therefore, anti-inflammatory treatment via inhibition of microglial activation is regarded as a promising strategy for preventing neurodegenerative diseases in the clinic.

Oxytocin (OT), a nonapeptide produced in the paraventricular and supraoptic nuclei of the hypothalamus, has a wide range of effects in the body. OT exerts its effects via G-protein-coupled receptors, which are expressed abundantly in the central and peripheral nervous systems [5]. OT plays a role in the endocrine and paracrine activities such as various sexual and maternal behaviors, social recognition, neuromodulation, cognition, aggression, and tolerance development [6]. Recent data indicate that OT may also have anti-inflammatory and anti-oxidant properties and regulate the immune and anti-inflammatory response [7, 8]. Exogenous OT administration alleviates tissue damage in a variety of animal models of injury [9–11]. Furthermore, co-administration of an OT receptor antagonist blocks the protective effects of OT during cardiac ischemia [10] or cerebral ischemia in rats [9]. The protective actions of OT in these models may be associated with decreased levels of circulating pro-inflammatory cytokines [12, 13] and decreased neutrophil infiltration to the site of injury [14, 15]. Moreover, OT inhibits LPS-stimulated pro-inflammatory cytokine secretion from macrophages and endothelial cells [16].

However, little information is available about the effects of OT on neuroinflammation and its underlying molecular mechanisms. Therefore, we aimed to investigate the anti-inflammatory effects of OT on LPS-stimulated microglial activation, and its therapeutic effects on the early stage of neuroinflammation induced by systemic LPS treatment in mice.

## Methods

### Cell culture

BV-2 cells retain many morphological and functional properties of primary microglia [17]. Cells in a 5 % CO_2_ incubator were maintained in Dulbecco’s modified Eagle medium (DMEM; Hyclone Co., Logan, UT, USA) with 10 % fetal bovine serum (FBS; Hyclone Co.), 2 mM L-glutamine, 100 U/ml penicillin, and 100 μg/ml streptomycin (Sigma-Aldrich, St Louis, MO, USA). For all experiments, BV-2 cells were used at 75–80 % confluency.

Primary microglia were prepared as described previously [18]. Briefly, the cerebral cortices of mice, aged 1–2 days, devoid of meninges and blood vessels, were dissociated by mild mechanical trituration. The isolated cells were cultured for 14 days in DMEM/F12 (Hyclone Co.) supplemented with 10 % FBS (Hyclone Co.). Then the mixed glial cultures were shaken on an orbital shaker at 250 rpm for 2 h to dislodge microglial cells. Cells were cultured for 7 days before treatment. The experimental protocol was approved by the National Institutes of Health Guide for Care and Use of Laboratory Animals (Publication No. 85-23, revised 1985), and efforts were engaged to minimize the number of animal usage and suffering.

Prior to use in the experiment, plated cells were incubated with serum-free DMEM for 1 h, and then the medium was replaced with serum-free DMEM containing either LPS (from *Escherichia coli*, serotype 0127:B8, Sigma-Aldrich) or OT (Sigma-Aldrich) for the various time intervals and concentrations as indicated below. OT was initially dissolved in normal saline. For most experiments, BV-2 cells and primary microglia were added 2 h before LPS (500 ng/ml) stimulation, while controls were treated with the vehicle (normal saline) except where indicated differently. This time point was chosen to minimize the possibility of any direct interactions between OT and LPS [9].

### Cell viability assay

BV-2 cells were seeded in 96-well culture plates at a density of 5 × 10^4^ cells/well. Cell proliferation was analyzed at 24 h after LPS pre-treated with or without differing concentration of OT, using the MTT assay. A volume of 20 μl MTT solution (5 mg/ml) was added to each well, and the cells were incubated for another 4 h in a humidified incubator at 37 °C. Then, after removing the supernatant, 200 μl of dimethylsulfoxide was added to each well and mixed thoroughly for 10 min. The optical density (OD) was measured at 490 nm. Cell viability was expressed as a percentage of viable cells obtained relative to that of controls.

### Immunofluorescence imaging

BV-2 cells and primary microglia were fixed in 4 % paraformaldehyde (PFA) for 20 min and blocked with 10 % goat serum in PBS. The glass slides with cells were incubated overnight in a humidified chamber at 4 °C with the following primary antibodies: ionized calcium-binding adapter molecule-1(Iba-1), 1:200, rabbit polyclonal (Wako); OT receptor (OTR), 1:500, goat polyclonal (Abcam, Cambridge, MA, USA). After primary antibody incubation, slides were washed and incubated with the appropriate fluorescent-conjugated secondary antibody (1:500 dilution, Sigma-Aldrich) for 1 h. Images were captured using a Nikon TE2000U microscope. The intensity of Iba-1 signal of each nucleus was counted at least six separate experiments by using Image-Pro Plus 6.0 software.

### Intracellular Ca^2+^ measurement

BV-2 cells grown on glass coverslips were washed three times with extracellular solution containing 150 mM NaCl, 5 mM KCl, 1 mM MgCl_2_ · 6H_2_O, 2 mM CaCl_2_, 1 mM glucose, and 10 mM HEPES (pH 7.4) and incubated with 1 μM Fura-2/AM for 40 min at 37 °C. Coverslips with Fura-2/AM-loaded cells were then mounted on a chamber positioned on the movable stage of an inverted microscope (Olympus IX70, Tokyo, Japan), which is equipped with a calcium imaging system (TILL Photonics). Fluorescence was excited at wavelengths of 340 nm for 150 ms and 380 nm for 50 ms at 1-s intervals by a monochromator (Polychrome IV), and the emitted light was imaged at 510 nm by an intensified cooled charge coupled device (TILL Photonics Image) through an X-70 fluor oil immersion lens (Olympus, Tokyo, Japan) and a 460-nm long-pass barrier filter. F340/F380 fluorescence ratio was recorded and analyzed with TILLVISION 4.0 software, which was used as an indicator of [Ca^2+^]i independent of intracellular Fura-2 concentration. The amplitude of [Ca2^+^]i response was defined as the peak of *F*/*F*0, where F0 is the average baseline fluorescence before the application of LPS (500 ng) and OT (1 μM), and *F* represents the fluorescence after the application of LPS and OT.

### Animals

BALB/C mice (adult male) weighing 22–25 g were obtained from Shandong University Animal Centre. All mice were housed in a 12-h dark/light cycle, temperature (20 ± 2 °C), and humidity-controlled environment with unlimited access to water and food. In the handling and care of all animals, the International Guiding Principles for Animal Research and Animal Research: Reporting In Vivo Experiments (ARRIVE) guidelines, as stipulated by the World Health Organization and as adopted by the Laboratory Animal Center at Shandong University, were followed. All efforts were made to reduce the number of animals used and their suffering.

### Nasal application of OT

Nasal application of OT was found to completely mimic the behavioral effects of OT seen after its intracerebroventricular administration [18, 19]. For nasal administration, mice received either OT (12 μg/2 × 6 μl) or vehicle (sterile Ringer solution, 2 × 6 μl) as previously described [19, 20]. Briefly, the amount of 12 μl was distributed with the tip of the pipette and allowed to diffuse into the squamous epithelium of both the left and right rhinarium, these area referred to as the glabrous skin around the nostrils which was highly innervated by free nerve endings [19, 20]. At the same time, to avoid direct contact of the tip of the pipette with the rhinarium, or direct application into one of the nostrils or in proximity of the philtrum, each of the applications to the left and right rhinarium, respectively, lasted about 1 min. To minimize non-specific stress responses, the experimental animals had 1 week of habituation to the holding position, as well as training to the procedure.

### Animal experimental protocol

A peripheral injection of LPS was administered to evoke neuroinflammation in mice as previously described [4]. LPS was freshly dissolved in sterile-endotoxin-free 0.9 % saline vehicle prior to injection. The LPS group (LPS) was intraperitoneally (i.p.) injected with a single dose of saline (5 mg/kg). In the control group, mice were injected i.p. with equivolume vehicle (0.9 % saline).

In group 1 (sham-operated group), equivolume vehicle (sterile Ringer solution) was nasal administered once 1 h prior to i.p. saline. In group 2 (sham + OT group), OT (2 × 6 μl) was nasal administered once 1 h prior to i.p. saline. In group 3 (LPS group), sterile Ringer solution was nasal administered once 1 h prior to LPS (5 mg/kg) injection. In group 4 (LPS + OT group), OT (2 × 6 μl) was nasal administered once 1 h prior to LPS (5 mg/kg) injection.

### Measurement of pro-inflammatory mediators

The prefrontal cortex of brain was removed from mice at 4 h after LPS injection (*n* = 5 at each group) for measure TNF-α and IL-1β messenger RNA (mRNA) levels by RT-PCR as described above.

The prefrontal cortex was removed from mice at 24 h after LPS injection (*n* = 10 each group) and homogenized with tissue protein extraction reagent (Pierce Biotechnology, Inc., IL, Rockford, USA) containing protease inhibitors, centrifuged at 12,000*g* for 10 min and the supernatant was collected to measure TNF-α and IL-1β content by Western blot analysis as described above.

### Tissue processing and immunofluorescence

Microglia activation in the brain tissue was observed with immunofluorescence. At 24 h after the LPS injection, the mice were deeply anesthetized, and the brains were fixed through cardiac perfusion with 4 % PFA, then dissected and post-fixed at 4 °C in 4 % PFA. The tissue sections (12 μm) were fixed in 4 % PFA for 10 min and blocked with 10 % goat serum in PBS. Slides were incubated overnight in a humidified chamber at 4 °C with the following primary antibodies: Iba-1(1:200, Abcam); TNF-α (mouse monoclonal, 1:200, Santa Cruz Biotechnology, CA, USA) and glial fibrillary acidic protein (GFAP)(rabbit polyclonal, 1:200, Abcam). After primary antibody incubation, samples were washed and incubated in the appropriate fluorescent-conjugated secondary antibody (1:600 dilution, Sigma-Aldrich) for 1 h. The slides were counterstained with DAPI for total nuclei counting. Images were captured with a Nikon TE2000U microscope. The microscope fields (×200) of Iba-1 positive cells or TNF-α/Iba-1, TNF-α/GFAP double positive cells in the prefrontal cortex from three different animals were randomly chosen and imaged. The frontal cortex was defined as the frontal region of the isocortex from the Bregma 5.5 to 1.0 mm, and it contained the primary and secondary motor cortices (analyzed at laterals 2.0 and 2.5) and the prefrontal cortex (analyzed at laterals 0.5 and 1.0; including orbitofrontal, cingulate, prelimbic and infralimbic cortices) [21]. The numbers of Iba-1 positive cells or TNF-α/Iba-1, TNF-α/GFAP double positive cells per field were calculated as the mean of the numbers obtained from the six pictures per mouse. The final data were reported relative to sham controls. Counting was performed in a blinded manner.

### Reverse transcription–polymerase chain reaction

Total RNA was extracted from cells and the prefrontal cortex using the Trizol reagent (Gibco, Invitrogen) according to the manufacturer’s instructions. RNA concentration was determined using a spectrophotometer (Bio-Rad. Labs) at 260 nm. Identical amounts of RNA (2 μg) were reversely transcribed into complementary DNA (cDNA) using a commercial reverse transcription–polymerase chain reaction (RT-PCR) kit (Fermentas, Vilnius, Lithuania) according to the manufacturer’s instructions. cDNA was subsequently amplified by PCR with specific primers. PCR products, separated on a 1.2 % agarose/TAE gel, were visualized by staining with ethidium bromide. The densitometric calculations of these values were normalized to β-actin. The intensity of bands was determined using Image-Pro Plus 6.0 software. The primers: TNF-α: *Forward (*5′- CGT CAG CCG ATT TGC TAT CT -3′*), Reverse (*5′- CGG ACT CCG CAA AGT CTA AG -3′*);* IL-1β: *Forward (*5′- AAG ATG AAG GGC TGC TTC CAA ACC -3′*), Reverse (*5′- ATA CTG CCT GCC TGA AGC TCT TGT -3′*);* iNOS: *Forward (*5′-CCT CCT CCA CCC TAC CAA GT-3′*); Reverse (*5′-CAC CCA AAG TGC TTC AGT CA-3′*);* COX-2: *Forward (*5′-TGG GTG TGA AAG GAA ATA AGG A-3′*); Reverse (*5′- GAA GTG CTG GGC AAA GAA TG-3′*);* OTR: *Reverse (*5′- TGG CCT TCA TCG TG TGC TGG A--3′*), Reverse (*5′- AGA GGA AGC GCT GCA CGA GTT--3′*),* β-actin: *Forward (*5′-TGG AAT CCT GTG GCA TCC ATG AAA C-3′*, Reverse (*5′- TAA AAC GCA GCT CAG TAA CAG TCC G-3′).

### Western blot analysis

Protein concentration of cells and the prefrontal cortex were determined using a BCA protein assay kit (Pierce Biotechnology, Inc.). A quantity of 20–40 μg of total proteins was loaded onto a 10–12 % gradient polyacrylamide gel, electrophoretically transferred to a polyvinylidene difluoride membrane, and probed with the following primary antibodies: TNF-α (1:1000, Santa Cruz), IL-1β (1:1000, Santa Cruz), phospho-NF-*κ*B p65(S536) antibody (1:500, Cell Signaling Tech. MA, USA), NF-*κ*B (1:1000, Cell Signaling), phospho-p38 antibody (1:1000, Cell Signaling), p38 antibody (1:1000, Cell Signaling), phospho-JNK antibody (1:1000, Santa Cruz Biotechnology, CA, USA), JNK antibody (1:1000, Santa Cruz Biotechnology), phospho-extracellular signal-regulated kinase (ERK)1/2 (1:2000, Cell Signaling), ERK1/2 (1:2000; Cell Signaling), OTR (1:2000, Abcam), inducible nitric oxide synthase (iNOS) (1:500, Cell Signaling), cyclooxygenase-2 (COX-2, 1:1000, Proteintech Group, Inc., CA, USA). β-actin (1:2000; Sigma-Aldrich) was used as an internal control. Secondary antibodies were horseradish peroxidase conjugated to goat/mouse anti-rabbit IgG (1:8000, Sigma-Aldrich). The membranes were developed using an enhanced chemiluminescence detection system (Pierce, Rockford, IL).

### Statistical analysis

Quantitative data were presented as the mean ± S.D. and statistical analysis of data was performed with a one-way ANOVA using the post hoc Tukey test for multiple comparisons of means. Differences were considered statistically significant if the *p* value was <0.05.

## Results

### OTR expression in BV-2 cells and primary microglia

**Fig. 1:**
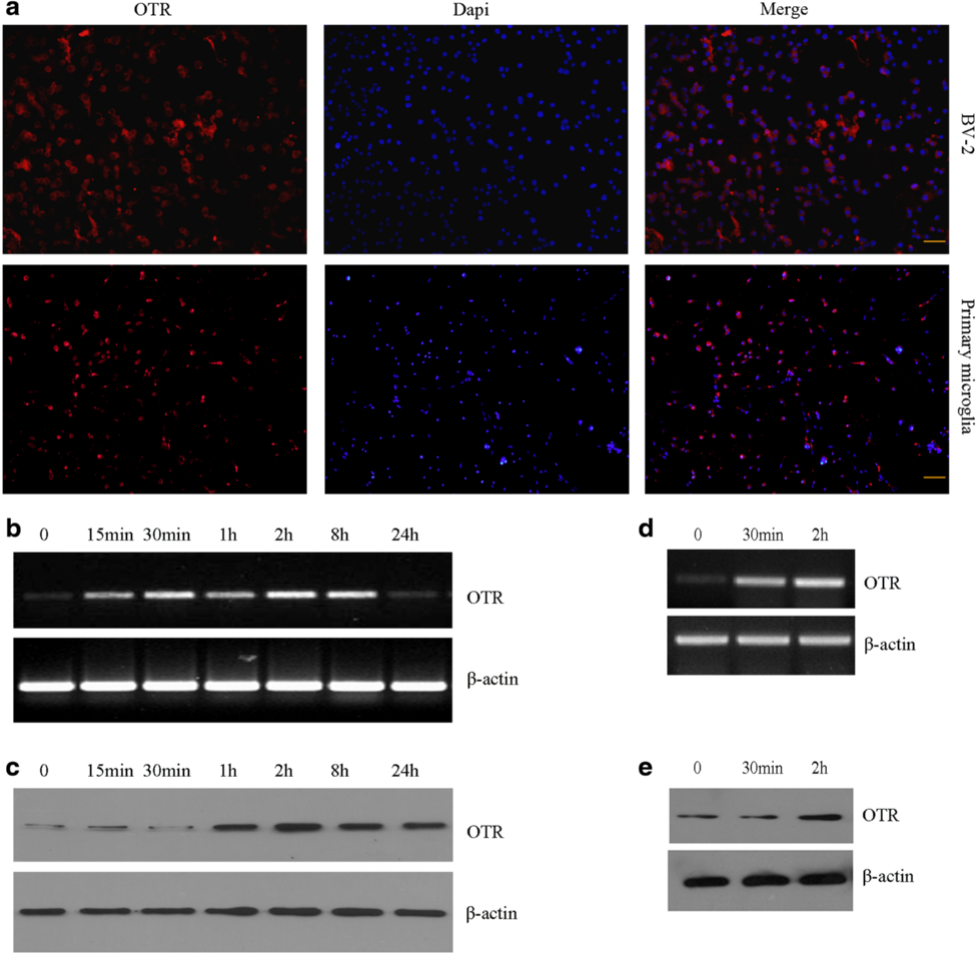
Oxytocin receptor (OTR) expression in BV-2 cells and primary microglia. **a** Immunostaining with anti-OTR (*red*) in BV-2 cells and primary microglia. Nuclei were counterstained with DAPI (*blue*). Scale bar = 50 μm. **b** OTR mRNA expression in BV-2 cells after LPS challenged were analyzed by RT-PCR, β-actin was used to evaluate loading. **c** OTR protein expression in BV-2 cells after LPS challenged was analyzed by Western blotting, and β-actin was used to evaluate protein loading. **d** OTR mRNA expression in primary microglia after LPS challenged were analyzed by RT-PCR, β-actin was used to evaluate loading. **e** OTR protein expression in primary microglia after LPS challenged was analyzed by Western blotting, and β-actin was used to evaluate protein loading

OTR expression was examined in BV-2 cells and primary microglia by immunofluorescence staining (Fig. 1a). OTR mRNA expressions were increased at 15 min after LPS challenge, reached the peak point at 30 min, and remained elevated at 8 h in BV-2 cells (Fig. 1b). OTR protein expressions measured by Western blot analysis were also increased at 60 min after LPS administration, reached the peak point at 2 h, and remained elevated at 24 h in BV-2 cells (Fig. 1c). Similar findings were obtained in primary microglia. The result showed that mRNA and protein expressions of OTR were increased after LPS treatment for 2 h in primary microglia (Fig. 1d).

To test the cytotoxicity of OT, various concentrations of OT were applied alone or together with LPS (500 ng/ml) to BV-2 cells for 24 h. Cell viability was determined by MTT assay. Normal untreated cells were considered as control. Cell viability following treatment with OT at 0.1 μM (106.12 ± 14.40 %), 1 μM (101.82 ± 13.24 %), and 10 μM (90.18 ± 8.98 %) was not significantly different from the control group (100 ± 11.99 %). LPS at 500 ng/ml cause a slight decrease in cell viability, but in comparison to control group the differences were not statistically significant (89.92 ± 13.80 % vs 100 ± 11.99 %, *p* > 0.05). And OT pre-treatment at 0.1 μM (88.17 ± 14.53 %), 1 μM (87.89 ± 13.90 %), and 10 μM (88.89 ± 13.03 %) was not significantly different from the LPS group (89.92 ± 13.80 %). Similar findings were obtained in primary microglia (data no shown).

### OT inhibits LPS-induced microglial activation

**Fig. 2:**
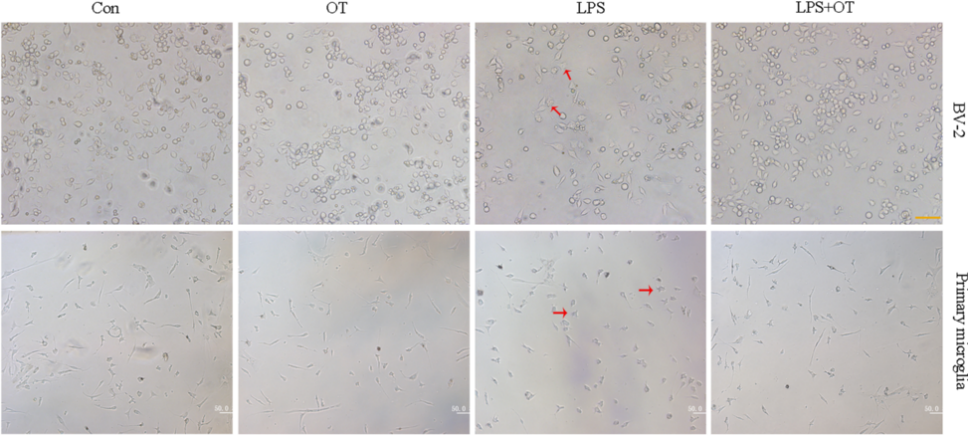
Effects of OT pre-treatment on LPS-induced morphological changes in BV-2 cells and primary microglia. Morphological changes of BV-2 cells and primary microglia per-treated with or without OT (1 μM) for 2 h followed by LPS (500 ng/ml) for 24 h. Note the change in external morphology of microglia bearing long extending and stout processes after LPS insult. Scale bar = 50 μm

Microglial cells are activated in response to different stimuli, which lead to morphological and functional changes. Therefore, to investigate whether the effect of OT on the morphology of microglia, cells were incubated for 2 h in the presence or absence of OT, followed by a 24-h LPS challenge. As shown in Fig. 2a, control cells are mostly round with bright refringency and small dark nuclei, whereas LPS treatment (500 ng/ml) induced an activated state presenting a rod-like bipolar or multipolar morphology with elongated cell bodies, and also showed ameboid. While pre-treatment of 1 μM OT in some extent helped to prevent this cellular transformation (Fig. 2). OT alone also had no significant effect on microglial cell morphology. Similar findings were obtained in primary microglia (Fig. 2).

**Fig. 3:**
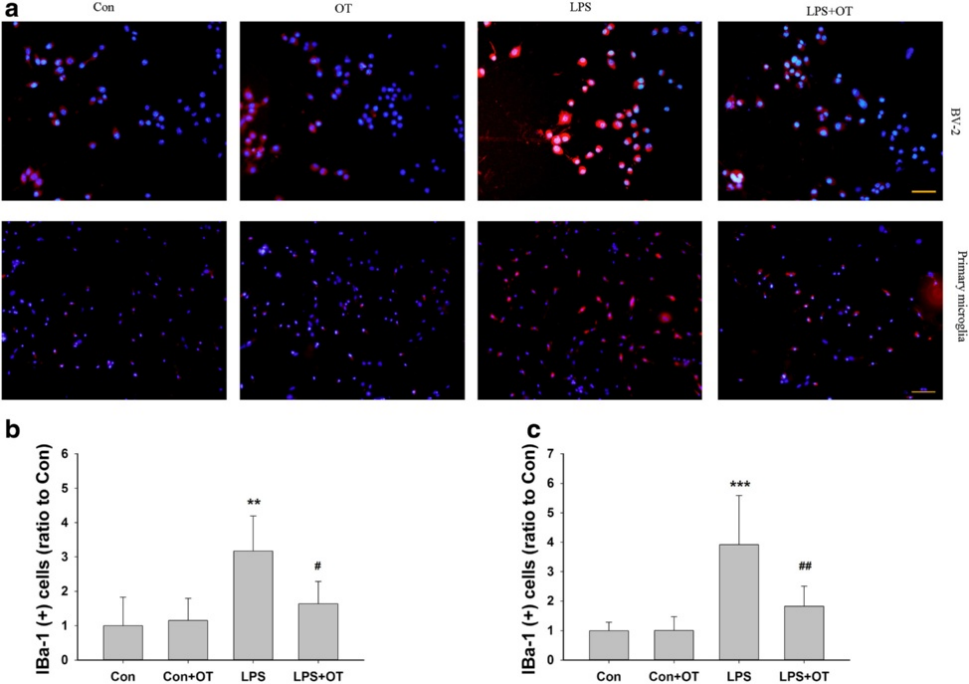
Effects of OT pre-treatment on LPS-induced microglial activation. **a** BV-2 cells and primary microglia were pre-treated with or without OT (1 μM) for 2 h followed by LPS (500 ng/ml) for 24 h, then stained with anti-Iba-1 (*red*), and counterstained with DAPI (*blue*). Quantification of the Iba-1 in BV-2 (**b**) and primary microglia (**c**) was determined by Image-Pro Plus 6.0. Scale bar = 50 μm. Images are representative of triplicate sets. Values were expressed relative to the fluorescence signal of respective controls. Values represent the mean ± S.D. **p < 0.01, ***p < 0.001 LPS VS Con; #*p* < 0.05, ##*p* < 0.01 LPS+ OT VS LPS

It had been reported previously that microglial activation was associated with a marked increase in Iba-1 expression. In our experiments, immunofluorescence analysis showed that at 24 h after LPS, the expression of Iba-1 was clearly increased in BV-2 cells (3.17 ± 1.02 vs 1.00 ± 0.83, *p* < 0.01) and primary microglia (3.92 ± 1.67 vs 1.00 ± 0.29, *p* < 0.005). Pre-treatment with OT (1 μM) attenuated this LPS-induced upregulation of Iba-1 in BV-2 cells (1.64 ± 0.65 vs 3.17 ± 1.02, *p* < 0.05) and primary microglia 1.82 ± 0.68 vs 3.92 ± 1.67, *p* < 0.01) (Fig. 3).

### OT suppresses TNF-α and IL-1β production in LPS-stimulated microglial cells

**Fig. 4:**
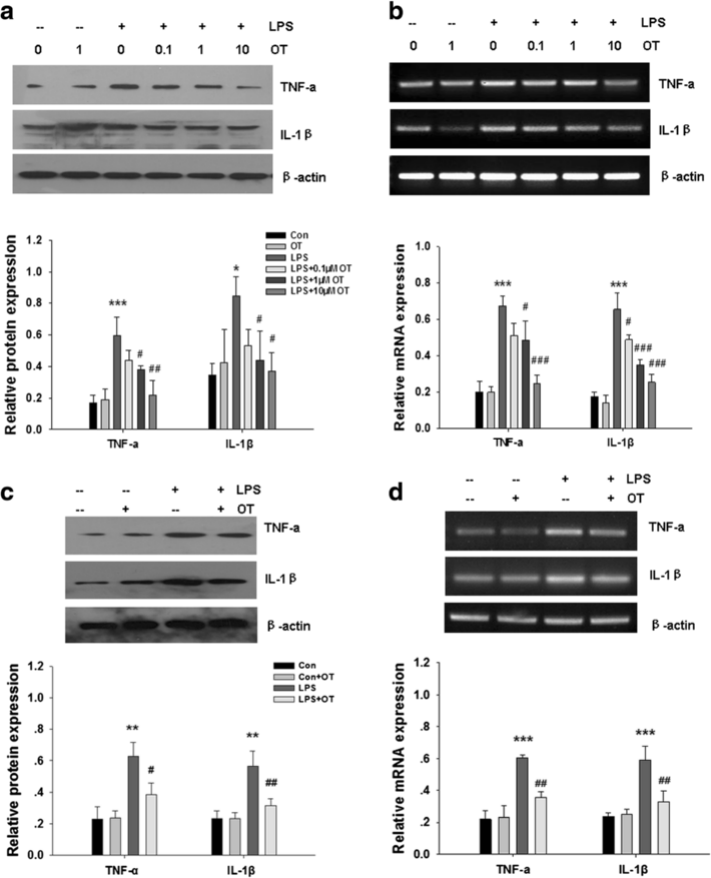
Effects of OT on LPS-induced pro-inflammatory cytokine mRNA expression and secretion in microglia. **a** BV-2 cells were pre-treated with or without OT (0.1–10 μM) for 2 h followed by LPS (500 ng/ml) for 24 h. The levels of pro-inflammatory cytokines were analyzed by Western blotting, and β-actin was used to evaluate protein loading. **b** BV-2 cells were pre-treated with or without OT (0.1–10 μM) for 2 h followed by LPS (500 ng/ml) for 4 h. The relative expression levels of TNF-α and IL-1β gene were analyzed by RT-PCR. **c** Primary microglia were pre-treated with or without OT (1 μM) for 2 h followed by LPS (500 ng/ml) for 24 h. The levels of TNF-α and IL-1β were analyzed by Western blotting, and β-actin was used to evaluate protein loading. **d** Primary microglia were per-treated with or without OT (1 μM) for 2 h followed by LPS (500 ng/ml) for 24 h. The levels of TNF-α and IL-1β mRNA were analyzed by RT-PCR, and β-actin was used to evaluate protein loading. Each value was normalized to β-actin. Values represent the mean ± S.D. of three independent experiments. *p < 0.05, *** *p* < 0.001 LPS VS Con; #*p* < 0.05, ##*p* < 0.01, ###*p* < 0.001, LPS + OT VS LPS

To assess whether OT could inhibit production of LPS-induced pro-inflammatory cytokines including TNF-α and IL-1β, BV-2 cells were pre-treated with OT for 2 h and then subjected to LPS for 24 h. As shown in Fig. 4a, LPS markedly increased TNF-α and IL-1β levels at 24 h post-stimulated with LPS. OT pre-treatment decreased LPS-stimulated TNF-α and IL-1β production showing a significant inhibitory effect at 1 and 10 μM, while OT (1 μM) alone had no effect on the production of TNF-α and IL-1β.

To elucidate the mechanisms responsible for the inhibitory effect of OT on TNF-α and IL-1β production, we next examined the levels of cytokine mRNA by RT-PCR. Consistent with the results obtained from the cytokine production data, the LPS-induced mRNA levels of TNF-α and IL-1β were reduced by OT (0.1, 1, and 10 μM) following 4-h treatment, suggesting that OT negatively regulated the production of TNF-α and IL-1β at the transcriptional level in these LPS-stimulated BV-2 cells (Fig. 4b).

The anti-inflammatory effects of OT were also observed in primary microglia. The results showed that OT (1 μM) pre-treatment significantly attenuated LPS-stimulated TNF-α and IL-1β protein and gene expression (Fig. 4c, d).

### Effects of OT on the expression of COX-2 and iNOS in microglial cells

**Fig. 5:**
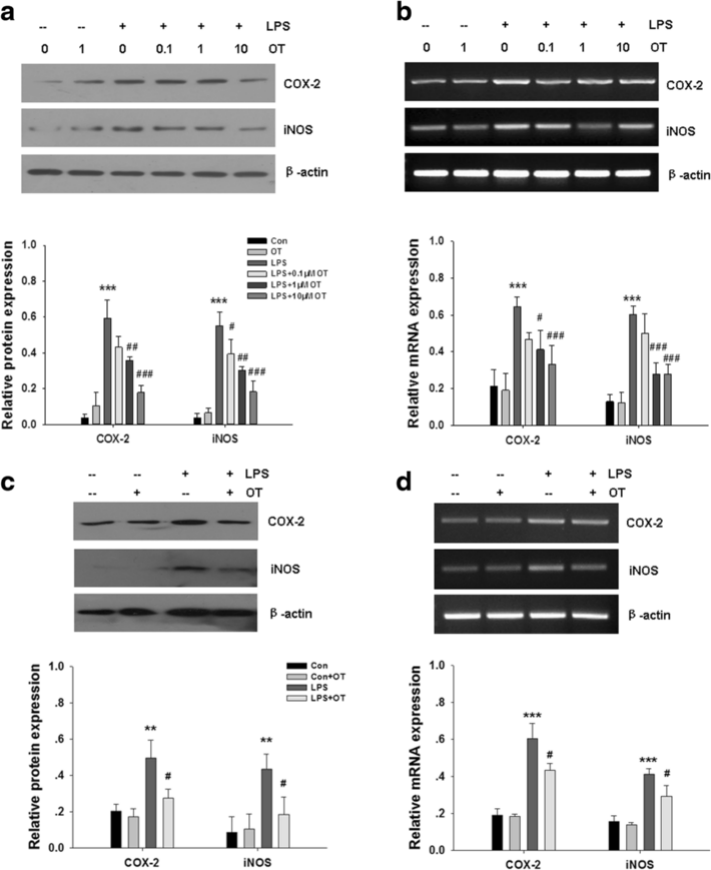
Effects of OT on LPS-induced COX-2 and iNOS mRNA and protein expression in microglia. **a** BV-2 cells were pre-treated with or without OT (0.1–10 μM) for 2 h followed by LPS (500 ng/ml) for 24 h. The levels of COX-2 and iNOS were analyzed by Western blotting, and β-actin was used to evaluate protein loading. **b** BV-2 cells were pre-treated with or without OT (0.1–10 μM) for 2 h followed by LPS (500 ng/ml) for 4 h. The relative expression levels of COX-2 and iNOS gene were analyzed by RT-PCR. **c** Primary microglia were pre-treated with or without OT (1 μM) for 2 h followed by LPS (500 ng/ml) for 24 h. The levels of COX-2 and iNOS were analyzed by Western blotting, and β-actin was used to evaluate protein loading. **d** Primary microglia were pre-treated with or without OT (1 μM) for 2 h followed by LPS (500 ng/ml) for 24 h. The levels of COX-2 and iNOS mRNA were analyzed by RT-PCR, and β-actin was used to evaluate protein loading. Each value was normalized to β-actin. Values represent the mean ± S.D. of three independent experiments. ***p < 0.001 LPS VS Con; #*p* < 0.05, ##*p* < 0.01, LPS+ OT VS LPS

We measured the changes of COX-2 and iNOS, which are key mediators in neuroinflammatory regulations. Western blot showed LPS stimulation significantly increased the expression of COX-2 and iNOS in BV-2 cells at 24 h post-stimulated with LPS. However, pre-treatment of OT could inhibit the protein expression of COX-2 and iNOS (Fig. 5a). Moreover, as shown in Fig. 5b, LPS significantly improved the mRNA levels of COX-2 and iNOS at 4 h post-stimulated with LPS and OT markedly reduced LPS-induced mRNA levels of COX-2 and iNOS in BV-2 cells.

Similar findings were obtained in primary microglia. The results showed that OT (1 μM) pre-treatment significantly attenuated LPS-stimulated COX-2 and iNOS protein and gene expression in primary microglia (Fig. 5c, d).

### OT reduced the increase in [Ca^2+^]_i_ in LPS-stimulated BV-2 cells

**Fig. 6:**
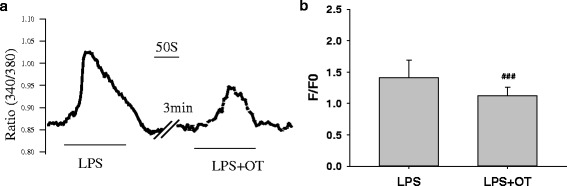
OT reduces the increase in [Ca^2+^]_i_ in LPS-stimulated BV-2 cells. **a** Representative 340/380 nm ratio of BV-2 cells showing the change of [Ca^2+^]_i_ in BV-2 cells as induced by LPS (500 ng/ml) in the absence or presence of OT (1 μM). **b** Summary data (*F*/*F*0) of LPS-induced [Ca^2+^]_i_ elevation, where *F*0 is the average baseline fluorescence before the application of LPS or OT, and *F* represents the fluorescence after the application of LPS or OT. *n* = 20 for LPS group; *n*  =  20 for LPS and OT-co-treated group, ###*p* < 0.001 LPS + OT VS LPS. All data were shown as mean ± S.D.

Previous studies showed that LPS could induce inflammatory responses and upregulate pro-inflammatory molecules through the action of calcium-independent mechanisms [22]. The intracellular calcium concentration ([Ca^2+^]i) was determined in all treatment groups using Fluo2-/AM assay. As shown in Fig. 6, the increase in [Ca^2+^]_i_ in BV-2 cells was remarkably reduced by OT (1 μM) (*p* < 0.001) treatment compared to LPS alone.

### Effect of OT on LPS-induced MAPK activation in BV-2 cells

**Fig. 7:**
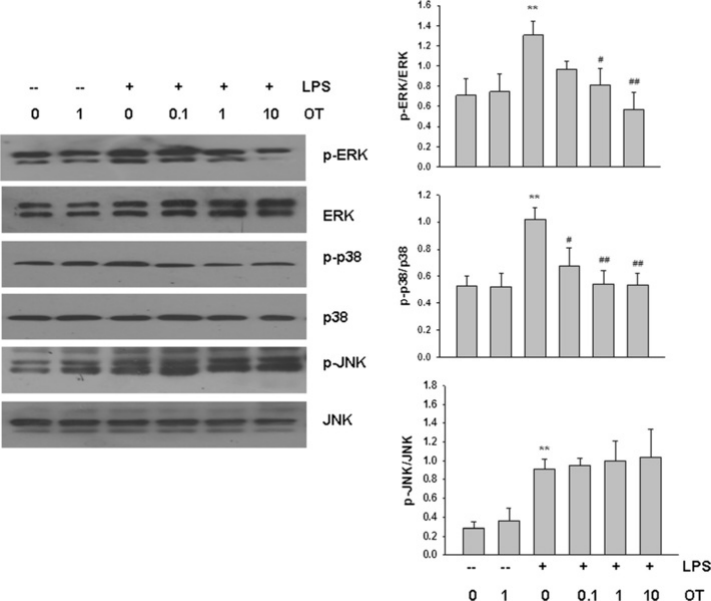
Effects of OT on LPS-induced phosphorylation of MAPK in BV-2 cells. BV-2 cells were pre-treated with or without OT (0.1–10 μM) for 2 h followed by LPS (500 ng/ml) for 60 min and total protein was subjected to Western blot analysis using antibodies against phospho-, or total forms of three MAPKs. Bar graphs showing quantification of expression levels of phosphor-MAPKs/MAPKs was determined by the Image-Pro Plus 6.0. Values represent the mean ± S.D. of four independent experiments. **p < 0.01 LPS VS Con; #*p* < 0.05, ##*p* < 0.01 LPS + OT VS LPS

We investigated the effect of OT on LPS-induced activation of mitogen-activated protein kinases (MAPKs), which are crucial in regulating the pro-inflammatory substances. LPS-treated BV-2 cells in the presence or absence of OT for 60 min were subjected to Western blot analysis. As shown in Fig. 7, pre-treatment OT (0.1, 1, 10 μM) significantly suppressed LPS-induced phosphorylation levels of ERK1/2 and p38 MAPK, while activation of JNK MAPK induced by LPS was not affected by OT.

### Effect of OT on LPS-induced NF-*κ*B activation in BV-2 cells

**Fig. 8:**
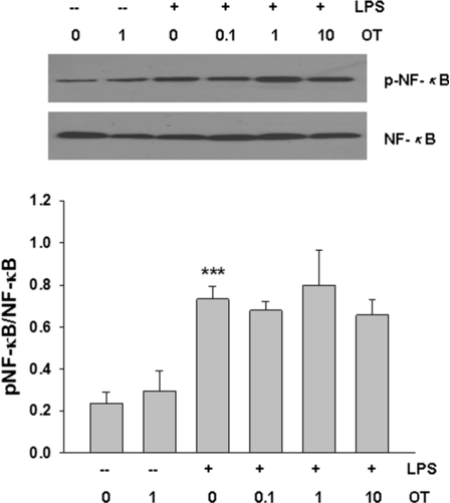
Effects of OT on LPS-induced NF-*κ*B activation in BV-2 cells. BV-2 cells were pre-treated with or without OT (0.1–10 μM) for 2 h followed by LPS (500 ng/ml) for 60 min and total protein was subjected to Western blot analysis using antibodies against I*κ*B-α, phospho-, or total forms of NF-*κ*B p65. Levels of β-actin were used to evaluate protein loading. Bar graphs showing quantification of expression levels of phosphor-NF-*κ*B (p-NF-*κ*B)/NF-*κ*B were determined by the Image-Pro Plus 6.0. Values represent the mean ± S.D. of three independent experiments. ***p < 0.001 LPS VS Con

We then assessed whether anti-inflammatory effects of OT on activated BV-2 cells were mediated via blockade of NF-*κ*B signaling pathway. So we examined the influence of OT on the NF-*κ*B phosphorylation by Western blot. Figure 8 illustrates that stimulation with LPS for 60 min induced significant NF-*κ*B phosphorylation. However, this effect was not affected by OT (0.1, 1, 10 μM) (*p* > 0.05).

### OT treatment is associated with a decrease of microglial activation in vivo

**Fig. 9:**
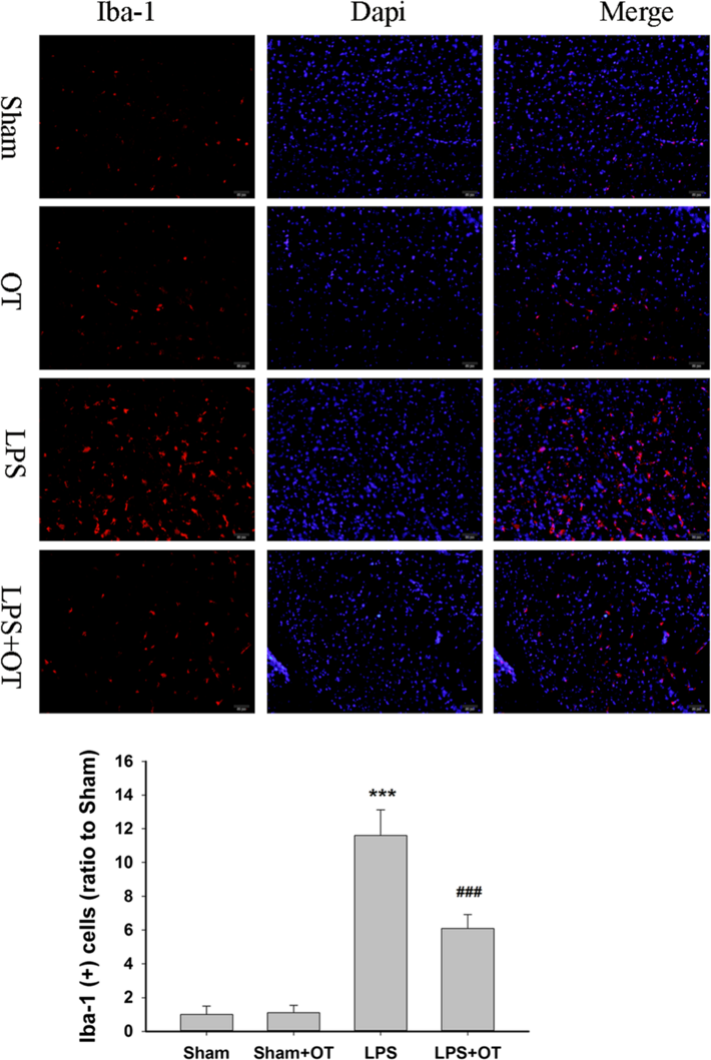
OT pre-treatment is associated with a decrease of microglial activation in vivo. Immunostaining with anti-Iba-1 (*red*) in the prefrontal cortex at 24 h after LPS treatment. Nuclei were counterstained with DAPI (*blue*). Scale bar = 50 μm. Values were expressed relative to the fluorescence signal of respective controls. Values represent the mean ± S.D., ***p < 0.01 LPS VS sham; ###*p* < 0.05 LPS + OT VS LPS

Systemic LPS treatment in mice is suggested to be able to cause microglial activation and neuroinflammation [4]. Next, we used the systemic LPS treatment acute inflammation murine brain model to study the suppressive effects of OT against neuroinflammation in vivo. The microglia exhibited enlarged cytoplasm and cell bodies, irregular shapes, and intensified Iba-1 staining, consistent with the morphological characteristics of activated microglia in the prefrontal cortex at 24 h after LPS treatment (Fig. 9). These effects were significantly reduced by OT pre-treatment. During this same developmental stage, OT did not influence microglial activity in the prefrontal cortex of sham controls.

### Effect of OT on TNF-α and IL-1β production in vivo

**Fig. 10:**
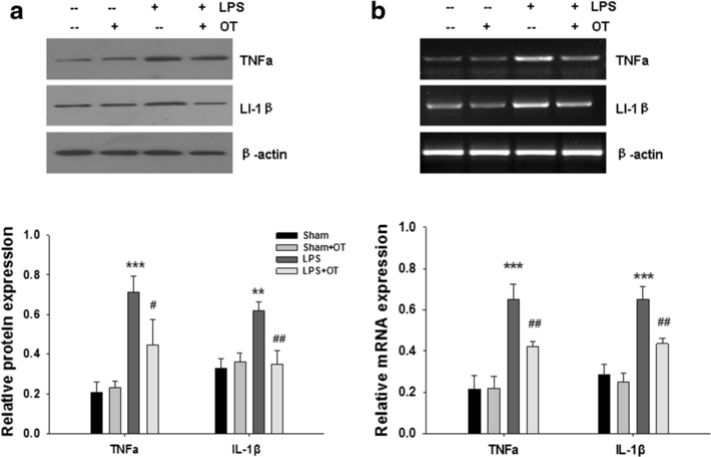
(**a**) The levels of TNF-α and IL-1β production in the prefrontal cortex at 24 h after LPS injection were measured by western blotting, and β-actin was used to evaluate protein loading. Data were obtained from three separate experiments. (**b**) The relative expression levels of pro-inflammatory cytokines in the prefrontal cortex at 4 h after LPS injection were analyzed by RT-PCR. Each value was normalized to β-actin. Quantification of mRNA levels of the various cytokines as determined by Image-Pro Plus 6·0. Data were obtained from three separate experiments, Values represent the mean ± S.D., ** *p* < 0.01, *** *p* < 0.001 LPS VS Sham; # *p* < 0.05, ## *p* < 0.01 LPS+OT VS LPS

Results from the Western blot assay showed that protein levels of TNF-α were increased significantly at 24 h after LPS exposure as compared with the sham group (*p* < 0.001; Fig. 10a). IL-1β protein levels were also increased significantly (*p* < 0.01) at 24 h after LPS exposure as compared with the sham group. These increases in TNF-α and IL-1β were reduced significantly with OT treatment when compared with LPS-treated mice that had not received OT (Fig. 10a). There were no significant differences in TNF-α and IL-1β production between sham and sham + OT mice.

The expressions of TNF-α and IL-1β mRNA in the prefrontal cortex were also detected by RT-PCR analysis at 4 h after LPS exposure. TNF-α mRNA expression was significantly increased in the prefrontal cortex after LPS challenged as compared with the sham group (*p* < 0.001; Fig. 10b). Similarly, increases in IL-1β mRNA expression, that paralleled TNF-α mRNA at 4 h post-LPS exposure, were observed as compared with that of the matching controls (*p* < 0.001; Fig. 10b). Pre-treatments with OT significantly decreased the mRNA expression of these selected genes (*p* < 0.01; Fig. 10b). However, OT by itself did not affect the expression of TNF-α and IL-1β mRNA in the sham group.

**Fig. 11:**
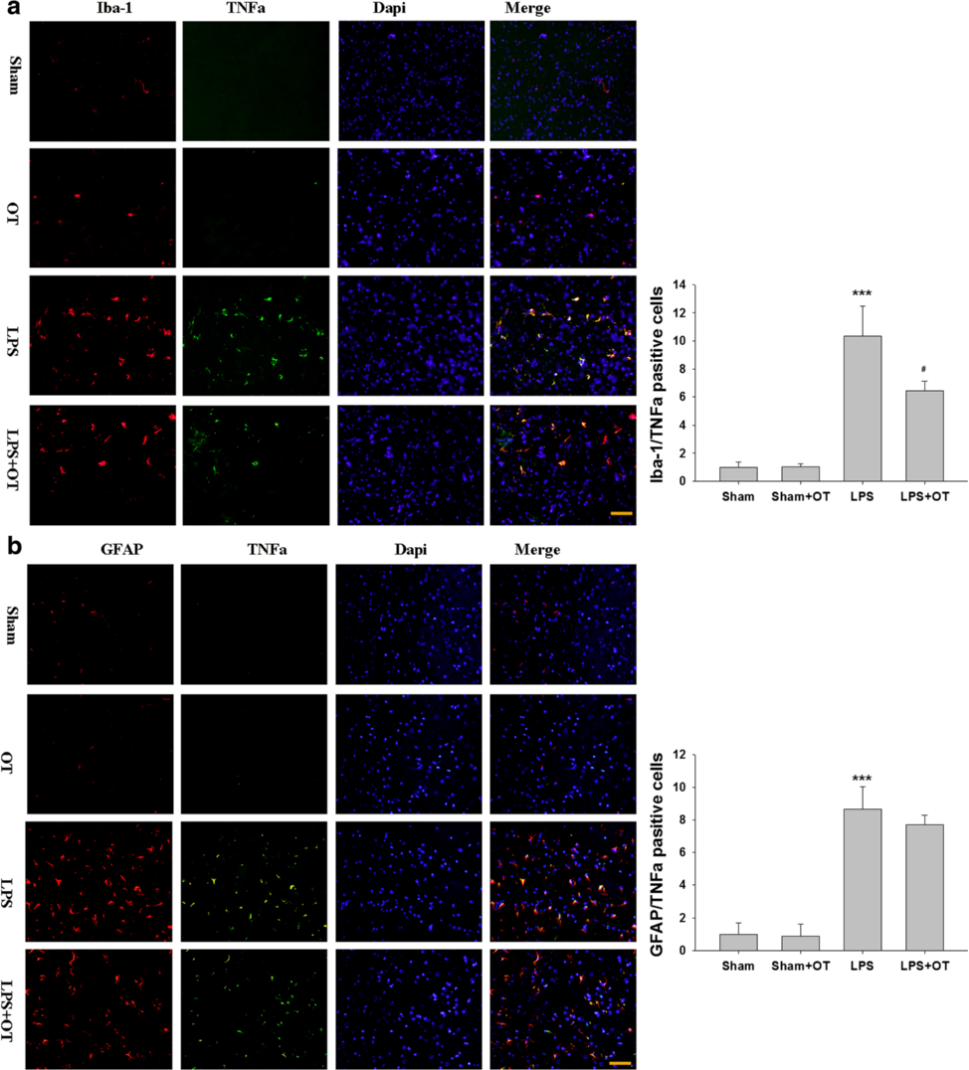
(**a**) Immunostaining with anti- Iba-1 (red) and TNF-α (green) in the prefrontal cortex at 24 h after LPS treatment. (**b**) Immunostaining with GFAP (red) and TNF-α (green) in the prefrontal cortex at 24 h after LPS treatment. Nuclei were counterstained with DAPI (blue). Scale bar = 50 μm. Values were expressed relative to the fluorescence signal of respective controls. Values represent the mean ± S.D., *** *p* < 0.001 LPS VS Sham; # *p* < 0.05 LPS+ OT VS LPS

In the prefrontal cortex of the sham group, TNF-α expression was specifically detected in sporadic cells, confirmed to be the microglia by double labeling with Iba-1 staining (Fig. 11a). At 24 h following LPS exposure, immunoreactivity for TNF-α/Iba-1 was detected and enhanced in large numbers of microglia when compared with the sham (Fig. 11). The statistical analysis showed that the percentages of TNF-α/Iba-1 double positive cells in the prefrontal cortex at 24 h following LPS exposure were significantly higher than those in the corresponding controls (*p* < 0.001, Fig. 11b). Moreover, these increases in TNF-α expression in microglia were reduced significantly with OT pre-treatment (*p* < 0.05, Fig. 11b).

The present results showed that the immunoreaction for TNF-α/GFAP was extremely weak in the prefrontal cortex of the sham group. While in the LPS challenge group, TNF-α/GFAP double positive cells showed strong hypertrophic and proliferative changes. The statistical analysis showed that the percentages of TNF-α/GFAP double positive cells in the prefrontal cortex at 24 h following LPS exposure were significantly higher than those in the corresponding controls (*p* < 0.001, Fig. 11b). And OT pre-treatment slightly decreased TNF-α/GFAP immunoreaction, but in comparison to LPS group, the differences were not statistically significant (*p* > 0.05, Fig. 11b).

### Effect of OT on COX-2 and iNOS expression in vivo

**Fig. 12:**
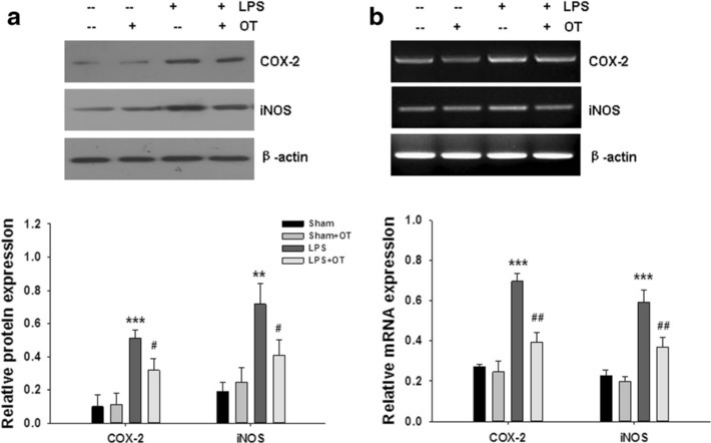
(**a**) The expression of COX-2 and iNOS in the prefrontal cortex at 24 h after LPS injection were measured by western blotting, and β-actin was used to evaluate protein loading. Data were obtained from three separate experiments. (**b**) The relative expression levels of COX-2 and iNOS in the prefrontal cortex at 4 h after LPS injection were analyzed by RT-PCR. Each value was normalized to β-actin. Quantification of mRNA levels of the various cytokines as determined by Image-Pro Plus 6·0. Data were obtained from three separate experiments, Values represent the mean ± S.D., ** *p* < 0.01, *** *p* < 0.001 LPS VS Sham; # *p* < 0.05, ## p < 0.01 LPS+OT VS LPS

Western blot showed systemic LPS treatment significantly increased the expression of COX-2 (*p* < 0.001) and iNOS (*p* < 0.01) in mice at 24 h post-stimulated with LPS. However, pre-treatment of OT was able to inhibit the protein expression of COX-2 (*p* < 0.05) and iNOS (*p* < 0.05) (Fig. 12a). As Fig. 12b shows, systemic LPS significantly improved the mRNA levels of COX-2 (*p* < 0.001) and iNOS (*p* < 0.001) at 4 h post-stimulated with LPS, and OT markedly reduced LPS-induced mRNA levels of COX-2 (*p* < 0.01) and iNOS (*p* < 0.01).

## Discussion

The present study demonstrated that OT suppressed the expression of TNF-α, IL-1β, COX-2, and iNOS at the mRNA and proteins levels and reduced the elevation of [Ca^2+^]_i_ in LPS-stimulated microglia cells. The activation of ERK and p38 MAPK by LPS was also suppressed by OT in vitro. Moreover, OT exhibited suppressive effects against neuroinflammation induced by systemic LPS treatment in vivo. These data suggested that OT would be a potential therapeutic agent for alleviating neuroinflammatory processes in neurodegenerative diseases.

Microglia-mediated neurotoxicity is a hallmark of the pathogenesis of various neurodegenerative diseases [23]. High levels of pro-inflammatory cytokines and chemokines released by excessive activated microglia are implicated in the process of neuronal injury [3]. Thus, therapeutic approaches targeting activated microglia may be a promising treatment of these diseases. Many recent studies have reported that anti-inflammatory agents exert their neuroprotective effects through inhibition of production of pro-inflammatory mediators [24, 25]. OT exhibited anti-inflammatory functions in rodents by reducing the secretion of TNF-α, IL-6, and IL-8 in vitro [16] and in vivo [26, 27]. In addition, OT regulated the immune response by dampening anti-inflammatory cytokines, such as IL-1ra and IL-4 [26]. Importantly, OT attenuated LPS-induced MHC Class II expression in cultured microglia [9]. In the current study, OT pre-treatment suppressed the LPS-induced gene and protein of TNF-α, IL-1β, COX-2, and iNOS levels in vitro and in vivo, showing a potential anti-inflammatory capacity in microglia.

Microglia are able to undergo a variety of morphological and functional changes in response to various immunological stimuli. Quiescent microglia exhibit a ramified cell morphology with relatively small size and numerous thin processes, whereas activated microglia become progressively less ramified and quickly develop an enlarged cell body with several short, thickened processes, resulting in a rounded ameboid-like appearance [4, 28]. In the present in vitro and in vivo study, we found that activated microglia were shown by increased cell size, irregular shape, shortened and thickened processes, and intensified Iba-1 staining in LPS-treated microglial cells and mouse brains, while OT lessened the microglia morphological changes according to immunohistochemical analysis. Moreover, OT significantly reduced Iba-1 protein expression in the brain compared to that of LPS group. Iba-1 is specifically and highly expressed in macrophage and microglia [29, 30]. Previous studies showed that anti-Iba-1 antibody was found to specifically recognize ramified microglia in normal rodent brain, and the expression of Iba-1 was strongly upregulated in activated microglia [31]. Therefore, Iba-1 may play significant roles in the regulation of some immunological and pathophysiological functions of microglia and serve as a novel marker of detecting the activation of microglia. Moreover, double immunofluorescence staining has shown that expression of TNF-α was detected in activated mciroglia as verified by their colocalization with Iba-1. In the current study, OT pre-treatment attenuated LPS-induced TNF-α/Iba-1 expression, indicating considerable inhibitory effects of OT on overactivated microglia via suppressing pro-inflammatory cytokine productions. In this study, we also saw LPS-induced neuroinflammation as evidenced by increases in TNF-α/GFAP immunostaining. However, OT had no effect on the LPS-induced increase in TNF-α/GFAP immunostaining, suggesting that anti-inflammatory effect of OT may be independent of the inhibition of astrocytic activation.

COX-2 and iNOS are the important inflammatory mediators during the inflammation process. COX-2, a key rate-limiting enzyme in arachidonic acid metabolism, is mainly induced in microglia by pro-inflammatory stimuli and plays important roles in inflammatory and immune responses [32, 33]. Several studies have demonstrated that COX-2 is markedly upregulated in primary brain microglia and in BV-2 microglial cells after LPS treatment [34, 35]. iNOS is expressed in some pathophysiological conditions and can produce abundant NO in response to inflammatory signals, such as LPS and cytokines [35]. Therefore, attenuating the induction of COX-2 and iNOS in activated microglia could inhibit neuroinflammation. In our study, LPS induced an increasing expression of COX-2 and iNOS in microglia. However, pre-treatment with OT showed an inhibition of COX-2 and iNOS protein and mRNA expression levels following LPS stimulation in vivo and in vitro.

Microglial cells express several receptors such as purinergic P2Y, adrenergic α1, thrombin, endothelin, platelet-activating factor, and cytokine/chemokine receptors, coupled to the second messenger inositol 1,4,5-trisphosphate IP_3_, and mediate release of Ca^2+^ from intracellular stores [36]. Ca^2+^ release often is followed by a prolonged secondary phase that is due to store-operated (capacitative) Ca^2+^ entry (SOCE). The intracellular calcium concentration ([Ca^2+^]i) influences multiple cellular functions, including enzyme or release activities. Microglia respond to CNS damage by upregulating functions that involve Ca^2+^ signaling, e.g., proliferation, migration, phagocytosis, and production of NO, IL, cytokines, and chemokines. Several factors have been identified to regulate the Ca^2+^ level in microglial cells. Cytokines, such as TNF-α, IL-1β, and interferon gamma (IFN-γ), induce an increase in [Ca^2+^]_i_ in microglia [37], and LPS also increases the prolonged component of [Ca^2+^]i and this Ca^2+^ increase is a prerequisite for the release of NO and cytokines [38]. In the present study, in good agreement with these previous studies, we demonstrated that LPS exposure significantly induced Ca^2+^ transients in microglial cells, while OT could abolish LPS-induced increases in [Ca^2+^]_i._ and cytokines levels, indicating OT effects on [Ca^2+^]_i_ from microglia activation may contribute to its anti-inflammatory actions. However, the direct correlation between the change in intracellular Ca^2+^ and anti-inflammatory actions of OT has not been documented.

LPS, an important structural component of the outer membrane of Gram-negative bacteria, binds to Toll-like receptor 4 and evokes intracellular inflammatory signaling cascades including NF-*κ*B, MAPKs, and IL-1 receptor-associated kinase activation [39]. NF-*κ*B is known to upregulate the expressions of cytokines, chemokines, adhesion molecules, acute phase proteins, and inducible effector enzymes. NF-*κ*B is composed of several protein subunits, among which p65 has been extensively studied [40]. As the expression of these pro-inflammatory mediators is modulated by NF-*κ*B, blocking NF-*κ*B transcriptional activity may be an important target for treating inflammatory diseases. In line with these findings, the data of our study showed that inflammatory cytokine production and NF-*κ*B activation were increased in LPS-stimulated microglia. However, the NF-*κ*B activation by LPS was not suppressed by OT pre-treatment, suggesting that inhibition of pro-inflammatory factors by OT was not mediated through blockade of NF-*κ*B activation.

MAP kinases are also crucial in regulating the pro-inflammatory substances such as TNF-α, IL-1β, IL-6, iNOS, and monocyte chemoattractant protein-1 expression in LPS-stimulated microglia cells [41, 42]. Corroborating these findings, the present results showed that increased concentrations of pro-inflammatory cytokines resulting from LPS treatment were accompanied by phosphorylation of ERK, p38, and JNK MAPK in microglial cells. Of particular significance to the present report were the findings that OT pre-treatment prevented phosphorylation of ERK and p38 MAPK, but not JNK and, in this way, reduced the upregulation of pro-inflammatory cytokines. However, Rimoldi et al. reported that stimulation of OTR with OT is associated with EGFR transactivation and ERK activation in HEK293 cells, resulting in opposite effects on cell growth [43]. And treatment with OT prevented the lethal reperfusion injury of H9c2 cardiomyoblasts and increased ERK phosphorylation [44]. It is important to mention that administration of OT in vivo effectively improved autism spectrum disorder-like symptoms, associated with inhibited phosphorylation of ERK in lesioned medial amygdalae [45]. One of the reasons for these inconsistencies could be due to the different cells used as well as the stimulating condition.

In the past decade, the structure and function of the OTR system have been detected and investigated in CNS [5]. For example, experiments with primary cell cultures showed that OTR are localized both on hypothalamic neurons and astrocytes [46]. It has been reported that OT concentration was increased in the temporal cortex and hippocampus of Alzheimer brains, but was normal in all other regions examined [47]. The higher level of OTR methylation was associated with decreased functional coupling of amygdala, which involved in affect appraisal and emotion regulation [48]. The OT mRNA level was increased after acute immobilization stress exposed to rats [49]. In this study, we found that OTR was expressed in microglial cells, and the mRNA and protein levels of OTR were increased after LPS stimulation.

In this regard, it is important to note that two recent studies indicated that OT did not attenuate inflammatory cytokine production following LPS stimulation in healthy human monocytes or macrophages in vitro [26, 50]. However, Clodi et al. reported that OT attenuated the endocrine and cytokine activation following LPS administration in healthy humans in vivo [26]. Similar results were also reported by other authors, for example, Szeto et al. demonstrated that OT inhibited LPS-stimulated pro-inflammatory cytokines secretion from human cancer cell line in vitro [16]. In addition, in our study, OT can abrogate LPS-induced microglial activation and reduce subsequent release of pro-inflammatory factors in murine cell line and primary cell. There are a number of reasons for the inconsistencies in anti-inflammatory effects of OT. For example, the healthy human cells behave differently from human cancer cell line, perhaps being less sensitive to OT than human cancer cells, or due to differences in how OTR is expressed. Thus, it may limit OT as a human therapeutic agent. Another reason for the inconsistencies is dependent on specific micro-environmental conditions which could be not replicated in vivo. There are also different functions between microglia and macrophage. At last, different stimulating condition used in these studies is also the reason for these inconsistencies, such as the time of LPS and OT incubation, the concentration of LPS and OT.

There are several limitations to our study. First, this is preliminary evidence found in a mouse model. Moreover, the studies of OT’s anti-inflammatory properties mainly based on animal models and human cancer cell lines [16, 51]. As mentioned above, few studies have investigated healthy humans and/or their cells and the results were inconsistent with animal models and human cancer cell lines [26, 50]. Thus, additional research in healthy humans’ microglia is needed in the future work. Second, the direct correlation between the change in OTR and LPS actions in microglia has not been documented. Finally, the route, timing, and dosage of OT treatment need to be further elucidated.

## Conclusions

In conclusion, out study demonstrates that OT significantly attenuates overactivation of microglial cells and reduces expression levels of pro-inflammatory mediators and cytokines via inactivation of ERK/p38 MAPK signaling pathways. These data suggests that OT would be a potential therapeutic agent for alleviating neuroinflammatory diseases accompanied by activated microglia.

## Abbreviations

CNS: central nervous system
COX-2: cyclooxygenase-2
DAPI: 4′, 6-diamidino-2-phenylindole dihydrochloride
DMEM: Dulbecco’s modified Eagle medium
ERK: extracellular signal-regulated kinase
FBS: fetal bovine serum
GFAP: glial fibrillary acidic protein
IL: interleukin
iNOS: inducible nitric oxide synthase
LPS: lipopolysaccharide
MAPK: mitogen-activated protein kinase
MTT: 3-[4, 5-dimethylthiazol-2-yl]-2, 5-diphenyltetrazolium bromide sodium
NF-*κ*B: nuclear factor *κ*B
OT: oxytocin
PBS: phosphate buffered saline
PFA: paraformaldehyde
RT-PCR: reverse transcription–polymerase chain reaction
TNF-α: tumor necrosis factor-alpha
